# Bacterial Communities Associated with Crude Oil Bioremediation through Composting Approaches with Indigenous Bacterial Isolate

**DOI:** 10.3390/life12111712

**Published:** 2022-10-27

**Authors:** Nilita Mukjang, Thararat Chitov, Wuttichai Mhuantong, Verawat Champreda, Wasu Pathom-aree, Pachara Sattayawat, Sakunnee Bovonsombut

**Affiliations:** 1 Department of Biology, Faculty of Science, Chiang Mai University, Chiang Mai 50200, Thailand; 2 Department of Entomology and Plant Pathology, Faculty of Agriculture, Chiang Mai University, Chiang Mai 50200, Thailand; 3 Environmental Science Research Center (ESRC), Chiang Mai University, Chiang Mai 50200, Thailand; 4 Biorefinery and Bioproduct Technology Research Group National Center for Genetic Engineering and Biotechnology (BIOTEC), Thailand Science Park, Pathum Thani 12120, Thailand; 5 Research Center in Bioresources for Agriculture, Industry and Medicine, Chiang Mai University, Chiang Mai 50200, Thailand; 6 Research Center of Microbial Diversity and Sustainable Utilization, Faculty of Science, Chiang Mai University, Chiang Mai 50200, Thailand

**Keywords:** bioremediation, compost, petroleum, crude oil, biodegradation, Next-Generation Sequencing (NGS), organic waste

## Abstract

**Simple Summary:**

Crude oil contamination of soil has affected human health and the environment. Several approaches have been explored as a choice to alleviate such contamination. The microbial community is known to play a role in several activities in the soil, including the degradation of pollutants. Composting approaches are used in this sense to provide nutrients for soil microorganisms and to introduce an exogenous community of microorganisms, to further facilitate the process. In this work, we aim to investigate the role of the microbial community in degrading crude oil-contaminated soil using fruit-based composting. To make it more interesting, a newly isolated indigenous crude oil-degrading bacterium was also added to one of the composted treatments. The composted treatments showed high efficiencies of crude oil bioremediation at 78.1–83.84%, which was around 6–6.5 times higher than that of the contaminated soil without composting materials. The bacterial community analysis indicated that there was a significant difference in bacterial composition between the non-compost and composted treatments, confirming that different communities of microorganisms resulted in different degradation efficiencies. Altogether, the fruit-based composting approach is an effective method for crude oil bioremediation in soil.

**Abstract:**

In this study, we aim to investigate the efficiency of crude oil bioremediation through composting and culture-assisted composting. First, forty-eight bacteria were isolated from a crude oil-contaminated soil, and the isolate with the highest crude oil degradation activity, identified as *Pseudomonas aeruginosa*, was selected. The bioremediation was then investigated and compared between crude oil-contaminated soil (S), the contaminated soil composted with fruit-based waste (SW), and the contaminated soil composted with the same waste with the addition of the selected bacterium (SWB). Both compost-based methods showed high efficiencies of crude oil bioremediation (78.1% and 83.84% for SW and SWB, respectively). However, only a slight difference between the treatments without and with the addition of *P. aeruginosa* was observed. To make a clear understanding of this point, bacterial communities throughout the 4-week bioremediation period were analyzed. It was found that the community dynamics between both composted treatments were similar, which corresponds with their similar bioremediation efficiencies. Interestingly, *Pseudomonas* disappeared from the system after one week, which suggests that this genus was not the key degrader or only involved in the early stage of the process. Altogether, our results elaborate that fruit-based composting is an effective approach for crude oil bioremediation.

## 1. Introduction

Crude oil contamination has been a global problem, especially in petroleum-producing countries. Crude oil contamination of soil has raised both health and environmental concerns. Hydrocarbons are the main component of crude oil that can accumulate in the food chain and this leads to many negative health impacts for humans, such as nervous system damage, reproductive system failure, and cancer [1]. Moreover, the accumulation of these substances in the environment, even in low concentrations, can be harmful to environmental health. The accumulation of crude oil in the soil can negatively affect soil inhabitants and microorganisms crucial to soil quality and plant health [2], whereas its accumulation in water sources also harms aquatic life, increasing their developmental defects and mortality, as well as reducing their reproductive capacity and disease resistance [3].

Crude oil-contaminated soil can be remediated by chemical, physical or biological means. However, emphasis has been placed on biological methods, which provide a holistic approach to crude oil remediation and apply to various types of environments, while having fewer negative impacts than chemical and physical remediation methods [4]. *Pseudomonas* along with other microorganisms including *Bacillus*, *Corynebacterium*, *Flavobacterium*, *Micrococcus*, *Rhodococcus*, *Acinetobacter*, *Burkholderia*, *Nocardioides*, *Dietzia*, *Microbacterium*, *Arthrobacter*, *Cellulomonas*, and *Gordonia* have been reported to have the ability to degrade crude oil [5,6]. In places where native microorganisms cannot degrade such pollutants, the introduction of exogenous species can enhance degradation efficiency. However, exogenous microorganisms may have low survivability or low growth rates in the soil to which they are introduced [7]. The re-introduction of a greater number of indigenous degrading bacteria may be an option as they already dwell in the environment and, thus, should be able to adapt promptly. The re-introduction of *Enterobacter cloacae*, an indigenous crude oil-degrading bacterium, showed a 54% degradation efficiency of the total hydrocarbon content in crude oil [8]. Another approach to bioremediation is to employ composting. The main purpose of this strategy is to provide nutrients to indigenous degrading microorganisms through composting materials. Additionally, the exogenous species can be introduced into the soil together with compost [9]. This strategy has been demonstrated in several reports using different composting materials [10]. However, there is insufficient knowledge about how bacterial communities change during bioremediation processes, nor is there sufficient information about the role of exogenous microbes, especially since different composting materials contain different microbial compositions. A few reports have investigated the bacterial community in different composting materials. For example, animal manures were used to co-compost with crude oil waste sludge and the bacterial community analysis showed that *Bacillus*, *Lysinibacillus*, *Microbacterium*, *Burkholderia*, *Dietzia*, *Rhodococcus*, *Pseudomonas*, and *Paeniclostridium* were the dominant genera with 36.5–99.9% polyaromatic hydrocarbon reduction [11]. Another work on the use of food waste supernatant for composting bioremediation of long-chain crude oil revealed that *Acinetobacter* and *Aquabacterium* dominated in the community along with *Brevundimonas* and *Pseudomonas* with the resultant bioremediation efficiency of 49.3% [12]. Therefore, this suggested that even though different bacterial communities were introduced to the composting bioremediation depending on the types of composting materials, they contributed to efficient bioremediation. Thus, in this work, fruit-based materials were selected as they were widely available in the area and easily applied to compost. Moreover, the re-introduction of contaminated soil indigenous microbes in compost-based bioremediation is another underexplored question. If the compost-based approach is to provide more nutrients to the indigenous degrading bacteria, what if a greater number of these bacteria are introduced along with the composting materials? Therefore, this study aims to address these questions by examining the dynamics of microbial communities during a period of compost-based bioremediation, both without and with the re-introduction of a crude oil-degrading bacterium.

## 2. Materials and Methods

### 2.1. Isolation of Crude Oil Degrading Bacteria

Crude oil-contaminated soil was collected from a petroleum production site [13], with the courtesy of the Petroleum Development Centre in Northern Thailand. The sample was diluted to 10^−1^ in sterile distilled water and the suspension (5% (*v*/*v*)) was transferred to 100 mL of Luria Bertani (LB) broth containing 1% (*v*/*v*) crude oil and incubated in an orbital shaker incubator (120 rpm) at 37 °C for 48 h. After incubation, the samples were centrifuged at 5000 rpm for 10 min and the cell pellets were washed twice with 0.1 M phosphate buffer (pH 6.8), and subsequently inoculated into 100 mL Bushnell Haas broth supplemented with 1% (*v*/*v*) crude oil. After incubation at 37 °C for 48 h (120 rpm), a 100 µL-portion was spread on Bushnell Haas agar plates, which were incubated at 37 °C for 7 days. Colonies grown on the agar plates were examined for cell morphology and maintained as pure cultures on Nutrient agar slant at 4 °C or as cell suspension in 15% (*v*/*v*) glycerol at −20 °C.

### 2.2. Screening of Bacterial Isolates Capable of Crude Oil Degradation

The bacterial isolates obtained from crude oil-contaminated soil were primarily screened for their surfactant-producing activity using the drop collapse test modified by Youssef et al., 2004 [14,15,16,17]. The drop shape was observed after 60 s and scored from 0 to 4, where 0 represents a full drop shape and 4 represents the complete collapse of the drop. A negative control (Bushnell Haas broth without bacterial culture; drop collapse score = 0) and positive control (a commercial detergent; drop collapse score = 4) were included for comparison.

The isolates having drop-collapse scores of more than 0 were cultured in LB broth at 37 °C, with agitation at 120 rpm, until OD_600_ reached 0.4, which corresponded to the bacterial number ca. 1 × 10^9^ CFU/mL. This pre-enriched culture was then inoculated into Bushnell Haas broth containing 1% (*v/v*) crude oil and incubated at 37 °C with agitation at 120 rpm for 30 days. After incubation, the degrees of crude oil degradation by the bacterial isolates were measured using a gravimetric assay [18]. Bushnell Haas broth containing 1% (*v/v*) crude oil was used as a negative control. The crude oil residue from each sample was then weighed and the percentage of crude oil degradation was calculated.

### 2.3. Identification of Selected Crude Oil Degrading Bacterial Isolates

The selected crude oil degrading isolate (M07, in this study) was identified using *16S rRNA* gene sequencing, using primers 63F and 518R [19,20,21] encompassing V1–V3 regions. The PCR was carried out in a thermocycler (GeneAmp PCR System 9700, Applied Biosystems, Foster City, CA, USA), following the conditions described previously [22], and the PCR products were then sequenced commercially at First Base Laboratories, Malaysia using Sanger sequencing. The sequences were subjected to nucleotide BLAST in the NCBI nucleotide database for species identification and deposited at the DNA Data Bank of Japan (DDBJ).

### 2.4. Compost-Based Bioremediation of Crude Oil-Contaminated Soil

Crude oil-contaminated soil was composted with fruit-based organic waste (ground mixed fruit peels of apple, cantaloupe, Chinese pear, guava, mango, watermelon, and corn (corn peel and corncob)). The experiment was carried out in pots (Ø = 20 cm, height = 20 cm) for three different treatments, each in triplicates. The treatments included (1) 0.8 kg of only crude oil-contaminated soil (untreated soil; hereinafter referred to as S), (2) 0.8 kg of crude oil-contaminated soil mixed with 1 kg of organic waste (soil composted with organic waste; SW) and (3) 0.8 kg of contaminated soil mixed with 1 kg of organic waste and 5% (*v/w*) of selected crude oil-degrading bacterial culture (which, in this study, was an overnight culture of M07 in LB broth) (soil composted with organic waste with the addition of crude oil degrading bacterium M07; SWB). Each pot was placed inside a clear plastic bag which was loosely tied with a rubber band. To prepare the SWB treatment, the contaminated soil and composting materials were mixed in a plastic bag before the overnight liquid culture of *P. aeruginosa* M07 was poured into the bag at the ratio of 5 mL of culture per 100 g of the contaminated soil and composting material mixture. All treatments were incubated in a well-ventilated roofed area for 4 weeks, with the surrounding air temperatures between 28.0 and 32.5 °C. Each pot was given vigorous agitation once a week. Aerobic plate count was performed on nutrient agar plates, which were incubated at 37 °C for 48 h. The analysis was performed every week for 4 consecutive weeks.

### 2.5. Chemical Analyses of Soil Samples

The pH of the soil samples (prepared by mixing 1 g soil sample in 2.5 mL of 0.01 M CaCl_2_) was measured using a pH meter (Ohaus, starter 3100, Parsippany, NJ, USA) every week throughout the 4-week bioremediation.

Crude oil degradation in the soil treatments was analyzed using thin-layer chromatography-flame ionization detection (TLC-FID (IATROSCAN, MK-6s)). The analysis was performed by the Microbial Technology Service Center, Chulalongkorn University, Thailand, according to the method described by Maruyama and colleagues [23].

### 2.6. Microbiological Analyses of Soil Samples Subjected to Bioremediation Treatments

Bacterial communities in the soil from each treatment were analyzed using Amplicon-metagenome analysis based on *16S rRNA* gene sequencing. A representative portion of the soil samples (about 25 g) from the three treatments were collected throughout the 4-week bioremediation. Each genomic DNA sample prepared as described by Phetchara et al. [13] was subjected to amplification targeting at the *16S rRNA* gene using E785F and E1081R primers (specific to the 5 and 6 hypervariable regions in the prokaryotic *16S rRNA* gene) attached with tagged barcode sequences [24]. The PCR was performed and subjected to Next-Generation Sequencing (NGS) using the ION PGM^TM^ platform sequencer (Life Technologies, Carlsbad, CA, USA). The sequences were deposited to the NCBI Sequence Read Archive (SRA) (accession no. PRJNA734089). Raw sequence reads were initially cleaned by removing low-quality reads with a cutoff of 20 for the Phred quality score and trimming their tagged and primer sequences using FASTP [25]. Next, the remaining reads were trimmed, denoised, and clustered into Amplicon Sequence Variant (ASV) using DADA2 [26]. Taxonomic classification was performed using the consensus BLAST against the Silva database [27] using a minimum fraction of assignment cutoff of 0.5. Beta diversity analysis based on unweighted Unifrac distance was used to compare the similarity between the two communities. An ordination plot by Principal Coordinate Analysis (PCoA) was performed to visualize the data. The analysis workflow is illustrated in detail in Figure S1.

### 2.7. Statistical Analysis

Differences in bacterial numbers among the treatments were statistically analyzed using Tukey honest significant differences (TukeyHSD function) in R version 4.0.3 with a confidence level of *p* ≤ 0.05. The analysis of variance model (AOV function in R version 4.0.3) was used to identify the differences among the treatments in relation to crude oil degradation. The significant effects of treatments in regulating bacterial communities were calculated by permutational multivariate analysis of variance (PERMANOVA) using the number of permutations of 999.

## 3. Results

### 3.1. Isolation of Crude Oil Degrading Bacteria and Assessment of Crude Oil Degradation Capacity of Bacterial Isolates

Forty-eight bacterial isolates were recovered from crude oil-contaminated soil collected at the site of an oil well in the Fang oil field in Northern Thailand. Six isolates (M02, M07, M10, M16, S120, and S104) had comparatively high surfactant-producing activities (with drop collapse test scores of 2 and 3). These isolates were further analyzed for crude oil degradation capacity using a gravimetric assay, from which one isolate, M07, was found to have the highest ability to degrade crude oil (Table 1). This isolate was identified as *Pseudomonas aeruginosa*, based on its *16S rRNA* sequence (DDBJ accession no: LC633334), and was further used for the culture-assisted composted treatment (SWB treatment). It should be noted, however, that although this bacterium was isolated from the crude oil-contaminated soil, it should not be assumed as a representative of this oil field.

### 3.2. Compost-Based Bioremediation Treatments of Crude Oil Contaminated Soil and Microbiological Analyses

**Figure 1:**
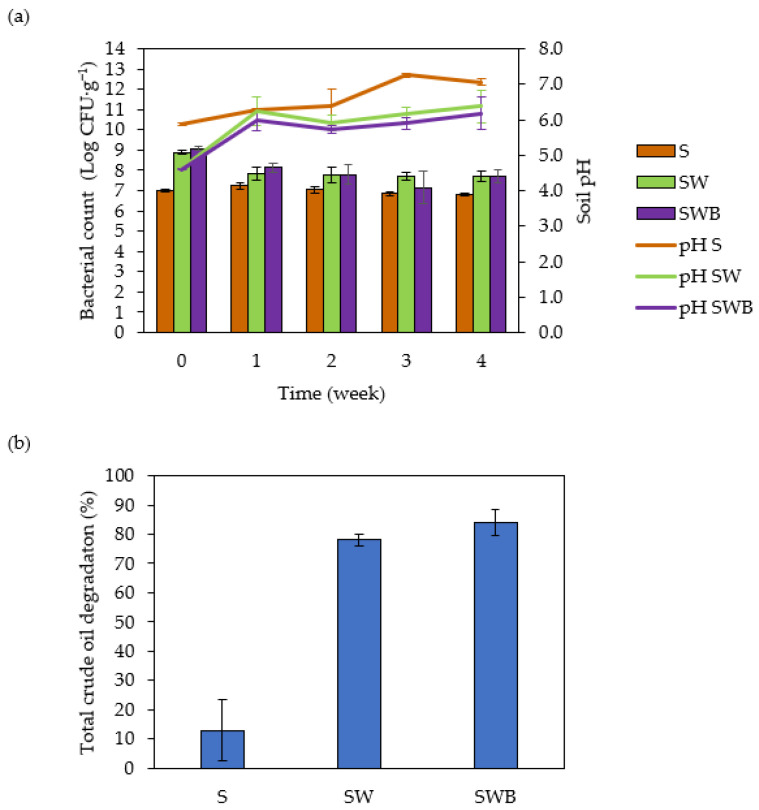
Compost-based bioremediation treatments of crude oil contaminated soil and microbiological analyses. (**a**) Changes in the bacterial number and soil pH during the 4-week period for the three treatments and (**b**) total crude oil degradation in three different treatments measured using TLC-FID. S: untreated soil, SW: soil composted with organic waste, SWB: soil composted with organic waste with the addition of crude oil degrading bacterium M07.

In order to evaluate how composting strategies could enhance the efficiency of crude oil degradation, bioremediation was carried out by means of composting with fruit-based waste, both without and with the addition of *P. aeruginosa* M07, hereafter SW, and SWB treatments, respectively. These compost-based bioremediation treatments were compared with the non-compost crude oil-contaminated soil (S treatment), which served as a control. Over the course of 4 weeks, the total bacterial count in the composted soil treatments (SW and SWB) decreased by approximately 1.3 log CFU/g (Figure 1a), and they were significantly different from the S treatment (TukeyHSD, *p* = 0.000038), but not significantly different (TukeyHSD, *p* = 0.9999) from each other. Although bacterial counts in both composted soils (SW and SWB) decreased during the bioremediation process, they remained higher than in the non-composted soil (S) by approximately 1 log CFU/g. Soil pH increased during the 4-week period in the untreated and the composted soils (Figure 1a), making the soil pH at the end of the bioremediation process to be 7 for the untreated soil and 6.4 and 6.2 for SW and SWB treatments, respectively.

Regarding total crude oil degradation in soil (analyzed using thin layer chromatography-flame ionization detection (TLC-FID)), it is clear that the compost-based treatments, both without and with the selected crude oil-degrading bacterium M07 (SW and SWB treatments), enhanced bioremediation efficiency by 6–6.5 times compared with bioremediation that relied on bacteria indigenous to contaminated soil alone (S treatment). At the end of the 4-week period, the SWB treatment showed the highest percentage of crude oil degradation at 83.84% (Figure 1b). The fruit-waste composted treatment (SW) achieved a crude oil degradation percentage of 78.11%, which was comparable with the use of corn waste as a compost material (77.56% within 8 weeks) [28], but this study showed a faster degradation rate for 4 weeks, while the study of Romanus et al. was conducted over the course of 8 weeks. The degradation in both composted treatments (SW and SWB) was significantly higher than in the untreated soil (S) (12.95%) (TukeyHSD, *p* = 0.0011 and *p* = 0.0007, respectively); however, they were not significantly different from one another (TukeyHSD, *p* = 0.8224).

### 3.3. Bacterial Community Analysis

**Figure 2:**
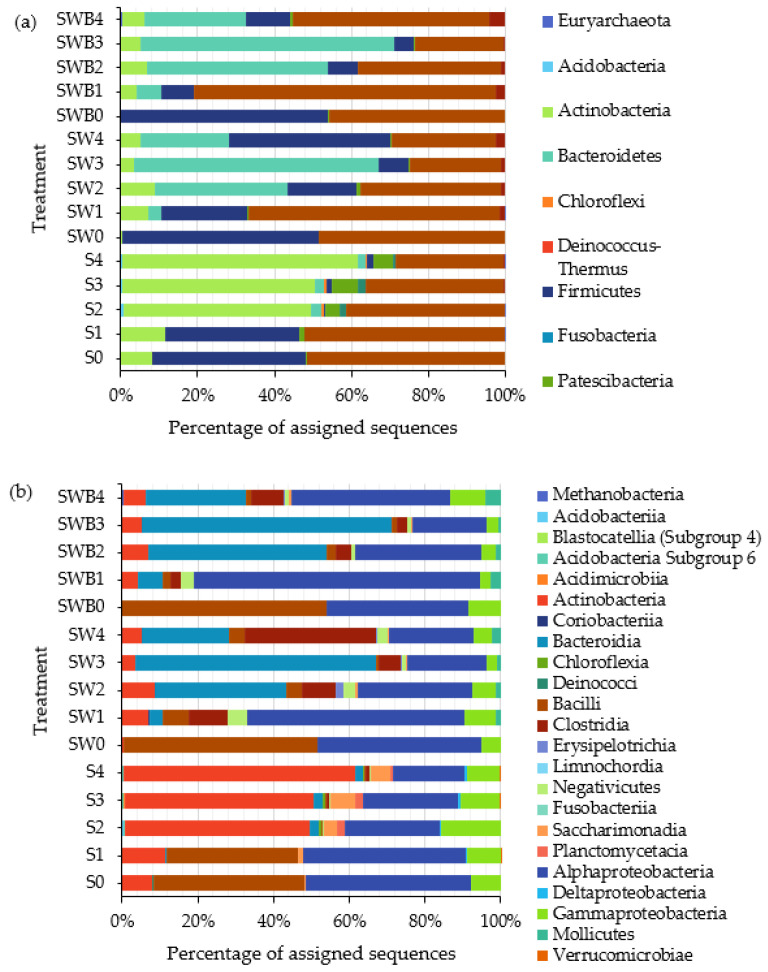
Bacterial communities at the phylum level (**a**) and the class level (**b**) in the soil of different treatments during the bioremediation period of 4 weeks. S: untreated soil, SW: soil composted with organic waste, SWB: soil composted with organic waste with the addition of crude oil degrading bacterium M07. The number following each treatment represents the week 0–4 of the bioremediation processes.

The bacterial communities in the soil of the three treatments were analyzed, using *16S rRNA*-based amplicon sequencing. In the crude oil-contaminated soil, Actinobacteria, Proteobacteria, and Firmicutes were the predominant phyla (Figure 2a). These are the phyla generally found in soil [29], including crude oil-polluted soil [30]. Overall, bacterial compositions differed significantly between composted and non-composted treatments. The most notable differences were the high relative abundance of Bacteroidetes and the low relative abundance of Actinobacteria in the composted soil (Figure 2a). In the untreated soil, the bacterial composition changed significantly during the second week by which Actinobacteria became dominant while Firmicutes and Proteobacteria decreased. In the composted soil treatments, Proteobacteria significantly increased during week 1 (seen in SW1 and SWB1, Figure 2a), and remained significantly abundant in both composted soil treatments. Bacteroidetes (mainly the class Bacteroidia) increased during weeks 2 and 3, and Firmicutes (mainly class Clostridia) increased during week 4 (Figure 2b). These bacterial community dynamics suggest that the bioremediation could be divided into two stages: a shorter first stage (weeks 0–1), when the bacterial compositions slightly changed from the starting point, and a longer second stage (weeks 2–4), when shifts in bacterial compositions became noticeable for all treatments.

Analysis of bacterial communities in the soils at the genus level revealed some predominant genera, including *Lactobacillus*, *Leuconostoc*, *Streptomyces*, *Acetobacter*, *Bifidobacterium*, and *Prevotella* (see Table S1 for details). Each had its distinctive dynamics in the non-composted and composted soils. In the untreated soil, between the second and the fourth weeks, *Streptomyces* became relatively more abundant, while *Lactobacillus* and *Leuconostoc*, became less abundant. In the SW and SWB treatments, *Acetobacter*, *Bifidobacterium*, and *Prevotella* increased in their relative abundances after one week. Although *Prevotella* became less abundant in week 4, it was still relatively high.

### 3.4. Beta-Diversity Analysis

**Figure 3:**
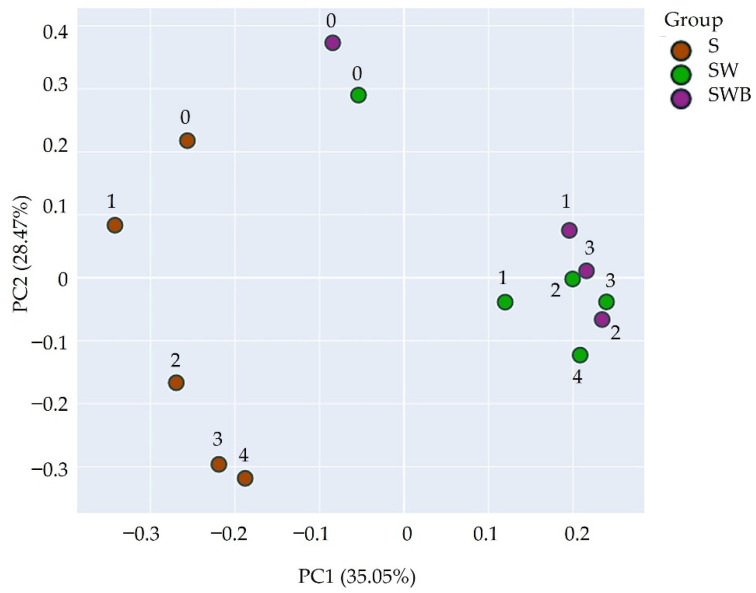
The differences among bacterial communities were measured through Beta-diversity analysis based on the unweighted Unifrac method. An ordination plot by Principal Coordinate Analysis (PCoA) was performed to visualize the data. The significant effects of treatments in regulating bacterial communities were calculated by permutational multivariate analysis of variance (PERMANOVA) using the number of permutations of 999. The microbial community of treatment S was significantly different from the other two treatments (*p* < 0.05).

Beta diversity analysis was used to visualize the difference in bacterial community structures between each treatment. The principal coordinate analysis (PCoA) in Figure 3 shows the Beta diversity of bacterial communities based on the unweighted Unifrac distance. The bacterial community in the non-composted soil differed significantly from the SW and SWB treatments (PERMANOVA, *p* = 0.009 and 0.008, respectively). In contrast, the bacterial communities in the SW and SWB treatments shared some similarities (PERMANOVA, *p* = 0.486). These results agree with the crude oil degradation study (Figure 1b), which also showed a significant difference between the S treatment and the other two treatments (SW and SWB). This observation suggests that the aforementioned predominant taxa of bacteria contributed either directly or indirectly to crude oil bioremediation.

## 4. Discussion

*Pseudomonas* is one of the well-known hydrocarbon degraders using the terminal oxidation pathway [31]. Recently, a member of this genus, *P. aeruginosa*, was isolated from an oil-contaminated lake wetland and reported to degrade up to 100% of C13–C35 hydrocarbons in crude oil [32]. In another work, this species was reported to degrade *n*-alkanes and polycyclic aromatic hydrocarbons at efficiencies of 80% and 98%, respectively [31]. In the present study, forty-eight isolates were obtained from crude oil-contaminated soil and *P. aeruginosa* was the isolate with a relatively highest level of crude oil degradation activity via a gravimetric assay. With this demonstrated potential, together with the reported literature, the newly isolated *P. aeruginosa* M07 was selected for composting bioremediation.

Our data demonstrated that the composted treatments resulted in 78.1 and 83.84% crude oil bioremediation efficiencies compared to 12.95% from the indigenous remediation. This could be because additional nutrients were provided to the soil microorganisms through composting materials. Therefore, it allowed the indigenous microorganisms to grow rapidly and degrade crude oil. Soil nutrients can be reflected by the carbon-to-nitrogen (C/N) ratio, which is one of the factors that can affect remediation efficiencies. Different C/N ratios in composted treatments have been demonstrated to result in different hydrocarbon degradation efficiencies [10]. In one study, the addition of mature compost to oily sludge resulted in a higher C/N ratio and an increase in hydrocarbon removal [33]. A C/N ratio of 10–40 was reported to be the most optimal regardless of any composting materials or conditions [10]. Food waste from markets and kitchens as a composting material has been characterized to have a C/N ratio of around 36 [34]. Though the C/N ratio of fruit-based waste used in this study has not been reported, it is anticipated to be similar to that of food waste. Interestingly, although *P. aeruginosa* M07 originated from an oil field, its addition to the SWB treatment did not result in a significantly higher crude oil degradation efficiency than the SW treatment. This is surprising but not wasted as it still evidences that both compost-based bioremediation approaches are efficient methods for remediating crude oil-contaminated soil. While it is difficult to directly compare degradation percentages measured over different intervals of time, the bioremediation using *P. aeruginosa* M07 (SWB) in our study also resulted in a higher degradation rate (83.84% within 28 days, ~3% per day) than an approach using agricultural wastes (wheat bran and swine waste) with a consortium of microorganisms (68.27% within 40 days, ~1.7% per day) [35] and better than the degradation in composting reactors with finished compost (86–92% within 84 days, ~1–1.1% per day) [36]. It must be noted, however, that these experiments were conducted on different scales and the properties of the crude oil might be different.

Surprisingly, *Pseudomonas* was not the predominant genus in the SWB treatment, even though a significant portion of this genus was found in week 0, this genus disappeared from week 1 onwards until the end of the composting period. This explains the insignificant difference in the bioremediation efficiencies between the two composted treatments as the main bacterial community compositions and dynamics were similar. This point is in contrast with a recent study where the indigenous *Pseudomonas* was found to be the most abundant genus in the composting treatment with different manures [11]. The disappearance of *P. aeruginosa* suggested that this species could not survive during the composting process, which brings us to the next question of which particular conditions may have such an impact on this species. Several abiotic factors can influence the composting process and, in turn, affect the microorganisms dwelling in the composting system. pH was one of the first abiotic factors considered in this study; however, a drastic shift in pH was not observed as the pH values ranged between 5–7 throughout the composting period, which is in the range of pH for *P. aeruginosa* growth [37]. The temperature was also considered an abiotic factor affecting the growth of *P. aeruginosa*, aerobic composting bioremediation is divided into 4 phases: mesophilic, thermophilic, cooling, and maturing phases [10]. The highest degradation rate is known to be discovered from the thermophilic phase and the temperature of this phase can be up to 70 °C [10], while *P. aeruginosa* can tolerate up to only 42 °C [38]. Although the size of the composting pots (Ø = 20 cm, height = 20 cm) in this study may not allow the temperature to be as high as 70 °C, especially when the ambient temperature was only around 30 °C, we anticipated that the change of temperature between each phase of the process may impact the growth conditions of *Pseudomonas*. Moreover, chemical components of the composting materials may also impact the microorganisms in the compost. Fruit peels are known to release antimicrobial compounds [39]. Some of the fruit peels used in this work have been reported to possess antimicrobial properties. Polyphenols from apple peels, for example, could inhibit the growth of a pathogenic bacterium, *Listeria monocytogenes* [40]. Similarly, water extracts of guava peels were also reported to inhibit the growth of *Staphylococcus aureus* and MRSA strains [41]. Watermelon peels have been shown to inhibit a number of microorganisms including *Pseudomonas fluorescens* [42]. Therefore, it should be noted that the antimicrobial properties of these fruit peels are another factor that is likely to impact the growth of some microorganisms present in the compost. Moving to biotic factors affecting the composting process, even though symbiotic and synergistic interactions of microbes, which naturally occur in a native soil environment, can support the growth or activities of indigenous species, the introduction of exogenous species through composting may result in the opposite outcome. Though the antagonistic behaviors of these microorganisms to the indigenous microbial community have not been elaborately reported, this factor should not be ruled out.

*Acetobacter* and *Prevotella* were the most abundant genera in the composted treatments; thus, they were assumed to play a key role in composting process. Although *Acetobacter* was present in week 0 in all treatments, the abundance decreased drastically from week 2 to week 3 in the untreated soil whereas its abundance remained relatively high in the composted treatments. These findings support our hypothesis that the addition of nutrients through composting materials supports the growth of indigenous microorganisms. Intriguingly, the addition of fruit-based waste allowed *Prevotella* to become one of the predominant genera in the bioremediation process. Even though this genus has been mostly associated with the human oral, gut, and respiratory tract [43], it has also been found in the residue and roots of rice plants [44] and cadmium-contaminated agricultural soil [45]. *Prevotella* has also been found as one of the predominant genera in the digestion reactor for biogas production using pig waste [46] and has been found to be suitable for diesel degradation [47]. Our finding further demonstrates the role of this genus in crude oil degradation. *Prevotella* was assumed to be introduced through the organic waste as the presence of this genus was not observed in week 0 of the untreated soil. This genus could be used as an example of the bacterial genera introduced via composting materials and end up playing an important role in the degradation process. Several studies suggest that the key players in composting bioremediation are the microorganisms introduced to the system via composting materials [48]. Altogether, the high abundance of these genera, *Acetobacter* and *Prevotella*, suggests that they were involved in the crude oil degradation process either directly or indirectly. *Acetobacter* is commonly found in petroleum hydrocarbon biodegradation processes together with other petroleum hydrocarbon degraders [49,50]. However, studies related to *Prevotella* have yet to be reported.

Beta diversity analysis allows clear visualization of the bacterial community structures and, as a result, the bacterial diversity of the untreated soil was significantly different from that of composted treatments. These differences suggest that the bacteria that play a key role in crude oil degradation were the ones introduced through the composting materials rather than the indigenous soil bacteria. Moreover, the similar structures between the two composting treatments emphasize that the exogenous bacteria predominated over *P. aeruginosa*.

Altogether, even though the re-introduction of indigenous *P. aeruginosa* did not significantly increase the bioremediation efficiency, the fruit-based composting approaches present in this study allowed 78.1–83.84% of crude oil to be degraded. The understanding of the microbial community from fruit waste that plays a role in crude oil bioremediation may be useful in order to provide a stepwise selection of composting materials. In particular, according to our results, *Prevotella* could be a potential genus in the community, especially since this work is the first report of its involvement in crude oil biodegradation. However, it should be noted that crude oil biodegradation may be a function of the community as a whole rather than the work of a single species. As this approach is easily applicable and eco-friendly, in the future, the system can be up-scaled even at crude oil production sites. In one study, full-scale composting bioremediation was applied to the petroleum-contaminated soil at an oil-polluted operational area using a mixture of sugarcane/bagasse as composting materials. After 2 months, up to 99% of crude oil was degraded [51]. Even though the preparation of the contaminated soil piles may be required prior to the composting, the application of this approach for crude oil degradation is considered relatively uncomplicated and environmentally friendly. Moreover, it is also noteworthy that the composting process emits a large number of gases such as CO_2_, CH_4_, N_2_O, and H_2_S [10]. These gases are a result of the decomposition of organic matter and microbial activities [52]. Several approaches, which have been summarized previously [52], are used to control the gas emissions from this process. The addition of additives, for example, is one of the strategies to mitigate gas emissions. These additives, such as phosphogypsum, are often added to either inhibit microbial activity [53] or to allow the transformation of these gases to other forms. The addition of bulk materials such as sawdust and straw for dairy manure composting has been shown to mitigate the emissions of CO_2_ and NH_3_ [53]. This is because the produced gases are absorbed by these materials, allowing a longer time for microbial assimilation of these gases. The introduction of microorganisms that can metabolize these gases in the compost is also another strategy to facilitate the assimilatory process. Nitrification is a reaction that converts NH_3_ to a nitrate; thus, the introduction of nitrifying bacteria has been shown to reduce the release of NH_3_ by 36% [54]. Compressing and covering the composting process has been shown to also reduce the aerobic respiration of microorganisms in the process, which results in a reduction in gas production. Finally, biofiltration of the gas released from the composting may be another promising strategy, though a closed system may be required for the effective implementation of this strategy [55].

## 5. Conclusions

This study focused on the bioremediation of crude oil-contaminated soil through compost-based approaches, particularly on exploring the potential roles of bacterial communities throughout the degradation processes. The differences in bacterial compositions between the non-composted and the composted treatments were immediately apparent and corresponded with differences in crude oil degrading efficiencies, although there was no significant difference in bacterial compositions and bioremediation efficiencies between the two composting methods. This suggests that the bacterial composition and other factors introduced through composting were more likely to contribute to the degradation of crude oil than *P. aeruginosa* M07. Some bacterial taxa that were predominant during the bioremediation processes were identified, including *Acetobacter* and *Prevotella*, implying a potential role for these taxa in the bioremediation of crude oil. The composting processes studied here were able to remediate crude oil in the contaminated soil up to about 78–84% in one month, making them one of the most efficient laboratory-scale compost-based bioremediation methods reported.

## Figures and Tables

**Table 1:** Drop collapse test and gravimetric analysis of crude oil degradation by biosurfactant-producing bacterial isolates obtained from crude oil contaminated soil at a petroleum production site in Northern Thailand.

Sample/ Isolate	Cell Morphology	Drop Collapse Score	Crude Oil Degradation (%) ^1^
Control ^2^	-	0	4.67 ± 2.69
M02	Gram-positive, rod-shaped	2	11.11 ± 6.42
M07 ^3^	Gram-negative, rod-shaped	2	21.56 ± 1.57
M10	Gram-positive, rod-shaped	2	11.30 ± 4.02
M16	Gram-negative, rod-shaped	2	7.44 ± 4.30
S104	Gram-positive, rod-shaped	3	12.71 ± 7.34
S120	Gram-negative, rod-shaped	2	10.37 ± 4.90

^1^ presented with standard error. ^2^ Control = Bushnell Haas Broth without bacterial isolate added. ^3^ M07 = the isolates with highest crude oil degradation activity, were identified as *Pseudomonas aeruginosa.*

## Data Availability

All the data associated with this research is included in this article and its supplementary information. Any further information is available upon reasonable request.

## Supplementary Materials

The following supporting information can be downloaded at: https://www.mdpi.com/article/10.3390/life12111712/s1, Figure S1: Analysis workflow of Next-Generation sequencing and bacterial community in this work. All pipelines and tools used in this study are mentioned; Table S1: Percentage of assigned sequence in the genus level in three different treatments.
