# Supplementary material for: Bacterial Communities Associated with Crude Oil Bioremediation through Composting Approaches with Indigenous Bacterial Isolate
Source: Life (Basel). 2022 Oct 27;12(11):1712. doi: 10.3390/life12111712 (PMC9692929; doi:10.3390/life12111712)
Supplement: Supplementary file 1 [file life-12-01712-s001.zip › life-1996132-supplementary.pdf]

Supplementary Materials

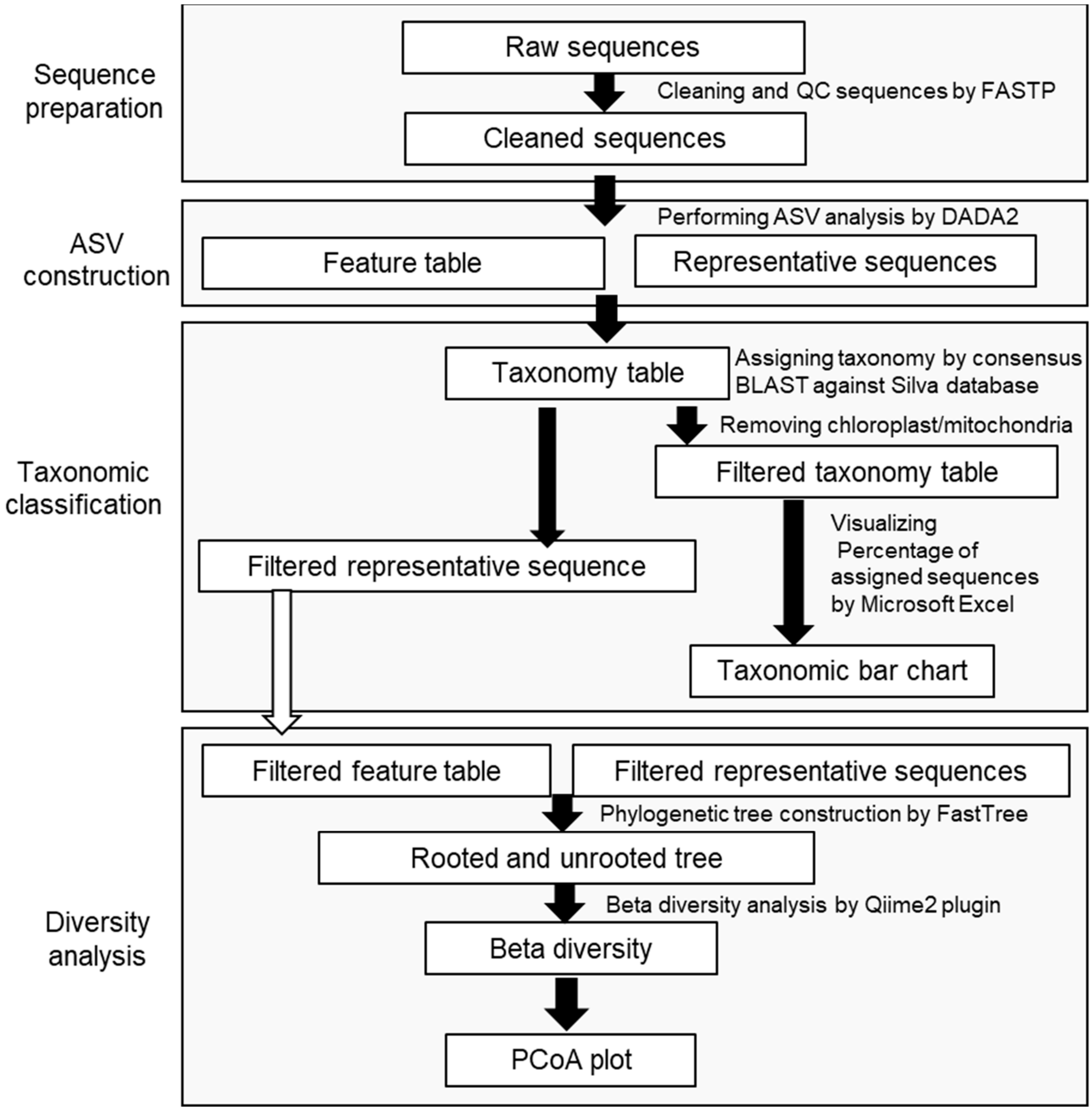

Figure S1. Analysis workflow of Next-Generation sequencing and bacterial community in this work. All pipelines and tools used in this study are mentioned.

Table S1. Percentage of assigned sequence in the genus level in three different treatments.

Genus	Percentage of assinged sequence in genus level in three different treatments [1]														
	**S0**	**S1**	**S2**	**S3**	**S4**	**SW0**	**SW1**	**SW2**	**SW3**	**SW4**	**SWB0**	**SWB1**	**SWB2**	**SWB3**	**SWB4**
Unassigned; Other	1.60	3.65	11.93	14.90	12.70	2.51	10.07	27.70	51.87	26.10	1.44	6.38	7.81	37.23	28.76
Acetobacter	41.26	38.00	2.98	1.65	3.65	42.95	58.26	29.18	18.53	19.81	36.97	74.93	32.75	18.64	35.13
Acinetobacter	0.30	0.13	0.00	0.35	0.56	0.14	1.49	0.28	0.00	0.00	0.08	0.00	0.00	0.00	0.36
Actinomyces	0.00	0.00	0.09	0.07	0.06	0.00	1.32	1.75	0.78	2.51	0.00	0.25	1.16	0.72	0.93
Actinophytocola	0.00	0.00	0.55	0.37	0.33	0.00	0.00	0.00	0.00	0.00	0.00	0.00	0.00	0.00	0.00
Amycolatopsis	0.67	0.72	3.92	3.21	1.69	0.00	0.00	0.00	0.00	0.00	0.00	0.00	0.00	0.00	0.00
Anaerocolumna	0.00	0.00	0.12	0.02	0.00	0.00	0.61	1.37	0.21	0.56	0.00	0.10	0.09	0.10	0.00
Anaerotruncus	0.00	0.00	0.00	0.07	0.03	0.00	0.10	1.08	0.80	1.07	0.00	0.00	0.56	0.55	0.80
Aquabacterium	0.00	0.04	0.34	0.23	0.25	0.00	0.00	0.00	0.00	0.00	0.00	0.00	0.00	0.00	0.00
Bacillus	0.68	0.36	0.18	0.00	0.09	1.34	0.13	0.25	0.00	1.04	0.67	0.00	0.00	0.00	0.00
Bifidobacterium	0.00	0.00	0.15	0.24	0.44	0.00	3.04	6.52	2.33	0.94	0.00	3.97	5.24	4.01	2.12
Bradyrhizobium	0.29	0.36	3.24	1.86	1.21	0.00	0.00	0.00	0.00	0.00	0.00	0.00	0.00	0.00	0.00
Burkholderia-Caballeronia-Paraburkholderia	0.11	0.21	0.94	0.71	0.36	0.00	0.07	0.00	0.00	0.08	0.00	0.00	0.00	0.00	0.00
Caproiciproducens	0.00	0.00	0.00	0.00	0.23	0.00	0.08	0.62	0.49	0.40	0.00	0.00	0.75	0.26	0.34
Christensenellaceae R-7 group	0.00	0.00	0.00	0.00	0.00	0.00	0.00	0.00	0.00	3.81	0.00	0.00	0.02	0.03	0.28
Clostridium sensu stricto 12	0.00	0.00	0.00	0.14	0.10	0.00	2.04	0.85	0.37	1.98	0.00	0.64	0.57	0.38	0.00
Cutibacterium	0.46	0.00	0.09	0.00	0.00	0.09	0.82	0.14	0.00	0.22	0.00	0.00	0.00	0.00	1.04
Dialister	0.00	0.00	0.00	0.07	0.04	0.00	0.96	0.83	0.74	0.63	0.00	0.55	0.53	0.80	0.83
Dokdonella	0.06	0.09	0.17	0.26	0.24	0.00	0.00	0.00	0.00	0.00	0.00	0.00	0.00	0.00	0.00
Dongia	0.12	0.16	1.13	1.54	1.17	0.00	0.00	0.00	0.00	0.00	0.00	0.00	0.00	0.00	0.00
Enterobacter	0.27	0.27	0.00	0.00	0.00	0.94	0.14	0.14	0.00	0.00	0.51	0.00	0.00	0.00	0.00
Fonticella	0.00	0.00	0.00	0.00	0.00	0.00	0.00	0.00	0.00	1.64	0.00	0.00	0.04	0.05	0.00
Gemmata	0.05	0.11	1.76	1.56	0.54	0.00	0.00	0.00	0.00	0.00	0.00	0.00	0.00	0.00	0.00
Gluconacetobacter	0.00	0.00	0.00	0.00	0.05	0.00	0.12	0.00	0.00	0.00	0.00	0.00	0.32	0.00	0.00
Gluconobacter	0.29	0.06	0.00	0.00	0.00	0.28	0.13	0.00	0.00	0.00	0.32	0.00	0.00	0.00	0.00
Herbinix	0.00	0.00	0.00	0.05	0.08	0.00	0.00	0.33	0.81	0.98	0.00	0.00	0.11	0.29	1.50
Immundisolibacter	0.21	0.57	2.60	0.91	0.95	0.00	0.00	0.00	0.00	0.00	0.00	0.00	0.00	0.00	0.00
Inquilinus	0.06	0.22	0.64	1.76	0.85	0.00	0.00	0.00	0.00	0.00	0.00	0.00	0.00	0.00	0.00
KCM-B-112	0.64	0.83	3.25	0.41	0.53	0.12	0.00	0.00	0.00	0.00	0.04	0.00	0.00	0.00	0.00
Klebsiella	0.45	0.19	0.00	0.00	0.00	0.91	0.08	0.00	0.00	0.00	0.63	0.00	0.00	0.00	0.00

[1] S: untreated soil, SW: soil composted with organic waste, SWB: soil composted with organic waste with the addition of crude oil degrading bacterium M07. The numbers following these abbreviations represent the week of composting processes.

Table S1. *Cont.*

Genus	Percentage of assigned sequence in genus level in three different treatments [1]														
	S0	S1	S2	S3	S4	SW0	SW1	SW2	SW3	SW4	SWB0	SWB1	SWB2	SWB3	SWB4
Komagataeibacter	0.22	0.14	0.06	0.10	0.10	0.25	0.28	0.21	0.16	0.18	0.06	0.16	0.06	0.05	0.00
Lachnoclostridium 5	0.00	0.00	0.00	0.03	0.06	0.00	1.03	0.07	0.00	0.46	0.00	0.91	0.09	0.00	0.00
Lactobacillus	29.02	25.51	0.00	0.22	0.23	27.21	5.37	2.13	0.09	0.58	39.04	1.95	0.68	0.35	0.00
Lactococcus	0.99	1.15	0.00	0.30	0.00	2.86	0.34	0.18	0.00	0.00	0.42	0.00	0.00	0.00	0.00
Leuconostoc	6.01	4.62	0.00	0.07	0.06	13.72	0.89	0.61	0.00	0.15	9.86	0.25	0.04	0.05	0.00
Luteimonas	0.04	0.08	0.31	0.28	0.23	0.00	0.06	0.00	0.00	0.00	0.00	0.00	0.00	0.00	0.00
Lysinibacillus	0.00	0.00	0.00	0.00	0.00	0.00	0.00	0.16	0.25	0.49	0.00	0.00	0.83	0.75	0.28
Lysobacter	0.38	0.92	2.16	2.25	1.49	0.00	0.00	0.00	0.00	0.00	0.00	0.00	0.00	0.00	0.00
Megasphaera	0.00	0.00	0.24	0.14	0.11	0.00	3.97	1.86	0.17	0.15	0.00	2.78	0.30	0.20	0.00
Microbacterium	0.48	0.92	5.31	2.52	2.10	0.04	0.05	0.00	0.00	0.00	0.00	0.00	0.00	0.00	0.00
Mycobacterium	3.13	3.55	12.26	9.16	7.09	0.05	0.00	0.00	0.00	0.14	0.00	0.00	0.11	0.16	0.00
Nocardia	0.44	0.85	3.77	1.82	1.58	0.00	0.00	0.00	0.00	0.00	0.00	0.00	0.02	0.00	0.00
Phenylobacterium	0.00	0.00	0.22	0.41	0.57	0.00	0.00	0.00	0.00	0.00	0.00	0.00	0.00	0.00	0.00
Prevotella	0.00	0.00	0.78	0.51	0.30	0.13	0.49	15.54	18.46	18.99	0.00	4.11	42.55	31.37	15.10
Promicromonospora	0.09	0.11	0.83	0.87	0.83	0.00	0.00	0.00	0.00	0.00	0.00	0.00	0.00	0.00	0.00
Pseudoclavibacter	0.00	0.00	0.00	0.00	0.01	0.00	0.05	0.54	0.23	1.79	0.00	0.00	0.13	0.06	0.98
Pseudomonas	2.00	1.69	0.21	0.22	0.05	0.00	0.00	0.00	0.00	0.00	5.75	0.00	0.00	0.00	0.00
Pseudoxanthomonas	0.38	0.98	3.37	2.01	1.30	0.00	0.00	0.00	0.00	0.00	0.00	0.00	0.00	0.00	0.00
Reyranella	1.53	3.57	12.17	12.20	6.59	0.00	0.00	0.00	0.00	0.00	0.00	0.00	0.01	0.04	0.00
Roseburia	0.00	0.00	0.00	0.07	0.07	0.00	1.53	0.00	0.00	0.12	0.00	0.00	0.04	0.00	0.00
Roseomonas	0.00	0.12	1.18	1.01	0.57	0.00	0.00	0.00	0.00	0.00	0.00	0.00	0.00	0.00	0.00
Ruminiclostridium	0.00	0.00	0.00	0.02	0.14	0.00	0.12	0.08	0.21	4.31	0.00	0.00	0.09	0.00	0.34
Ruminococcaceae UCG-010	0.00	0.00	0.00	0.00	0.04	0.00	0.00	0.00	0.00	1.18	0.00	0.00	0.00	0.00	0.00
Rummeliibacillus	0.00	0.00	0.00	0.02	0.02	0.00	0.00	0.33	0.16	1.25	0.00	0.00	0.61	0.15	1.14
Saccharopolyspora	0.00	0.00	0.21	0.35	0.56	0.00	0.00	0.00	0.00	0.00	0.00	0.00	0.00	0.00	0.00
Sedimentibacter	0.00	0.00	0.00	0.03	0.12	0.00	0.59	1.13	0.77	3.52	0.00	0.00	0.45	0.14	0.96
Serratia	1.64	1.13	0.40	0.29	0.26	0.07	0.00	0.00	0.00	0.10	0.00	0.00	0.02	0.00	0.00
Stenotrophomonas	0.44	0.43	0.32	0.28	0.26	0.27	0.41	2.07	1.07	2.40	0.13	0.00	1.67	0.65	4.66
Streptomyces	2.33	4.80	22.01	33.89	48.35	0.00	1.00	0.09	0.08	0.15	0.00	0.00	0.12	0.22	0.80
Sutterella	0.00	0.00	0.11	0.16	0.17	0.00	3.34	2.20	0.78	1.39	0.00	2.91	1.65	2.09	1.61
Terrimonas	0.00	0.10	0.00	0.41	0.25	0.00	0.00	0.00	0.00	0.00	0.00	0.00	0.00	0.00	0.00
Weissella	3.39	3.34	0.00	0.00	0.00	6.11	0.31	0.40	0.00	0.19	4.07	0.07	0.09	0.04	0.00
Xanthomonas	0.00	0.00	0.00	0.00	0.36	0.00	0.70	1.39	0.60	0.67	0.00	0.05	0.51	0.62	2.02

[1] S: untreated soil, SW: soil composted with organic waste, SWB: soil composted with organic waste with the addition of crude oil degrading bacterium M07. The numbers following these abbreviations represent the week of composting processes.
